# High Repetition Rate Mid-Infrared Differential Absorption Lidar for Atmospheric Pollution Detection

**DOI:** 10.3390/s20082211

**Published:** 2020-04-14

**Authors:** Yu Gong, Lingbing Bu, Bin Yang, Farhan Mustafa

**Affiliations:** 1 Collaborative Innovation Center on Forecast and Evaluation of Meteorological Disasters, Key Laboratory for Aerosol-Cloud-Precipitation of China Meteorological Administration, Key Laboratory of Meteorological Disasters, Ministry of Education, Nanjing University of Information Science and Technology, Nanjing 210044, China; 20181205001@nuist.edu.cn (Y.G.); farhan@nuist.edu.cn (F.M.); 2 Nanjing Institute of Advanced Laser Technology, Nanjing 210038, China; ybin319@163.com

**Keywords:** convolution correction, mid-infrared, differential absorption lidar, high repetition rate

## Abstract

Developments in mid-infrared Differential Absorption Lidar (DIAL), for gas remote sensing, have received a significant amount of research in recent years. In this paper, a high repetition rate tunable mid-infrared DIAL, mounted on a mobile platform, has been built for long range remote detection of gas plumes. The lidar uses a solid-state tunable optical parametric oscillator laser, which can emit laser pulse with repetition rate of 500 Hz and between the band from 2.5 μm to 4 μm. A monitoring channel has been used to record the laser energy in real-time and correct signals. Convolution correction technology has also been incorporated to choose the laser wavelengths. Taking NO_2_ and SO_2_ as examples, lidar system calibration experiment and open field observation experiment have been carried out. The observation results show that the minimum detection sensitivity of NO_2_ and SO_2_ can reach 0.07 mg/m^3^, and 0.31 mg/m^3^, respectively. The effective temporal resolution can reach second level for the high repetition rate of the laser, which demonstrates that the system can be used for the real-time remote sensing of atmospheric pollution gas.

## 1. Introduction

Presently, monitoring pollutants in the environment is crucial for preventing chemical poisoning and disease onsets. Polluting, toxic, flammable and explosive gases have been widely concerned for the public health and security. The release of a large amount of chemical substances in the environment has a harmful impact on human health and safety. Several industrial plants use dangerous chemicals for compounds synthesis. An accidental release of these chemicals in the environment can easily lead to serious accidents [1]. Furthermore, several harmful gases are important components of “Chemical warfare”, which can seriously endanger public safety [2,3]. Regular monitoring and quantification of polluting, toxic, flammable and explosive gases is inevitable to ensure the public health and security.

As an important means of monitoring atmospheric gases, DIAL has many advantages over other conventional detection instruments, such as good continuity, large detection range, high measurement sensitivity etc. It is widely used in multiple fields including atmospheric, agricultural and industrial gas detection. At present, great progress has been made in the visible and ultraviolet spectral regions DIALs, which can determine and quantify the concentration of various gases in the atmosphere [4,5,6,7,8]. Ultraviolet DIAL uses Dye laser or Raman laser. The laser structure is complex and the wavelength tuning range is narrow, thereby, limiting detection capability. Compared to the UV DIAL, the research on the infrared spectral region, especially the mid-infrared spectral region, is relatively scarce. However, the spectrum of mid-infrared (2.5–25 μm) is mainly produced by the vibration of molecular fundamental frequency. Many atmospheric components and dangerous gases such as CH_4_, SO_2_, NO_2_, NO, H_2_S etc. have strong absorption capacity in the mid-infrared spectral region, their absorption spectrum is often hundreds to thousands of times stronger than those in the near infrared region [9]. Therefore, mid-infrared lasers have good application prospects in the field of gas component detection [10,11]. In recent years, a series of achievements have been made in the research of gas detection in the mid-infrared band. S. Veerabuthiran et al. [12] developed a 3.0–3.45 μm DIAL system with a laser energy of 5 mJ, line width less than 7 cm^−1^ and frequency of 10 Hz. The designed system could be used to measure the concentration of thiodiglycol (TDG) vapor plumes in open state. The maximum detection distance was 1 km, and the sensitivity was about 10 ppm·m. D. Mammez et al. [13] presented a 1.98–2.30 μm DIAL system. The laser has energy of 5–10 mJ, line width of 60 MHz, and repetition rate of 30 Hz. The system could realize the concentration distribution measurement of H_2_O, CO_2_ and CH_4_. The detection sensitivity of CH_4_ was about 2 ppm. S. Lambert-Girard et al. [14] developed a 1.5–3.8 μm DIAL system, with laser energy of 15–17.5 μJ, laser line width of 10–200 nm, which could be used to detect H_2_O, CO_2_ and CH_4_ simultaneously, with a minimum detection accuracy of 1 ppm·m. O.A. Romanovskii et al. [15] developed a 1.8–2.5 μm and 3–4 μm DIAL system, with laser energy of 9–25 mJ, laser line width of 2 cm^−1^, and repetition rate of 30 Hz, which could be used to detect H_2_O, CO_2_. The error in recovering the concentration from the lidar measurement data did not exceed 0.4 g/m^3^ for H_2_O and 0.13 ppm for CO_2_.

The OPO DIAL lidar systems described above work either in the near or in the middle IR region of the spectrum. All the discussed systems obtained high laser energy but the repetition rate was relatively low.

The aim of this work is to develop a high repetition rate 2.5–4 μm DIAL system and evaluate its performance for detection of atmospheric pollution plumes at long distances. The system can be used for monitoring the emission of industrial polluted gases and the environment around chemical plants. We used techniques, such as convolution correction, monitoring channel to reduce system error. Taking NO_2_ and SO_2_ as the experimental objects, gas cell calibration experiment and the open field observation experiment were carried out to verify the performance of the equipment.

## 2. DIAL Theory

Different gas molecules have their own characteristic absorption spectrum. The principle of DIAL detection is according to the difference in the absorption capacity of the laser at different wavelengths. DIAL emits two laser beams of different wavelengths. One beam is at the strong absorption position of the target gas named as λ_on_, and the other beam is at the weak absorption or non-absorption position named as λ_off_. The concentration of the target gas can be measured by detecting the difference between the returned signals of the two lasers [16]. Assuming the transmitted power is *P_t_*, the overall system efficiency is η, the telescope receiving area is A, the distance of the hard target is *R*, the reflectivity of the hard target is *ρ*, the absorption cross-sections of λ_on_ and λ_off_ are σ_on_ and σ_off_, the atmospheric attenuation coefficient is α and the number concentration of the molecule is *N(R)*, then the energy *P* received by the detector can be expressed as [17,18,19]:

(1) $$P=P_{t}\eta \frac{A}{R^{2}}\rho \cdot exp\left\{-2\int_{0}^{R}\alpha +N\left(R\right)\cdot \sigma_{\mathrm{on},\mathrm{off}}dR\right\}.$$

According to the inversion method of DIAL, after some algebraic manipulations, add the Avogadro constant *N_A_* and the gas molecular mass *M*. Then *N(R)* is converted into the standard unit (mg/m^3^). The average gas concentration on the path *R* can be expressed as:

(2) $$N\left(R\right)=-\frac{1}{2R\Delta \sigma }\left[\;ln\left(\frac{P_{\mathrm{on}}}{P_{\mathrm{off}}}\right)\right]\cdot \frac{M}{N_{A}}.$$

where ∆σ is the differential absorption cross-section (∆σ = σ(λ_on_) − σ(λ_off_)) [20]. *P*_on_ is the echo signal of λ_on_ and *P*_off_ is the echo signal of λ_off_. The premise of using Formula (2) for the concentration inversion is that the parameters of λ_on_ and λ_off_ are consistent except for wavelength. However, it is difficult to guarantee in practice. Considering the inversion problem caused by the deviation of initial energy, divergence angle and coaxial of λ_off_ and λ_on_ etc., we use a mid-infrared detector in the lidar system to record the initial laser energy of λ_on_ and λ_off_ as *P*_on1_ and *P*_off1_ to correct the system deviation. Finally obtains the DIAL inversion equation [21]:

(3) $$N=-\frac{1}{2\Delta \sigma R}\left[\ln\left(\frac{P_{\mathrm{on}}\cdot P_{\mathrm{off}1}}{P_{\mathrm{off}}\cdot P_{\mathrm{on}1}}\right)\right]\cdot \frac{M}{N_{A}}.$$

The sensitivity of the DIAL method is characterized by the minimum measurable concentration of the gas molecule that can be detected with the minimum errors in optical signal. The expression for the minimum detectable content is given below [12]:

(4) $$N_{min}=\frac{1}{2\Delta \sigma R}\left[\ln\left(1+\left(\frac{1}{SNR}\right)\right)\right].$$

where *N_min_* is the detection sensitivity of target gas and *SNR* is the detection signal-to-noise ratio of the system.

## 3. DIAL System Description

**Figure 1:**
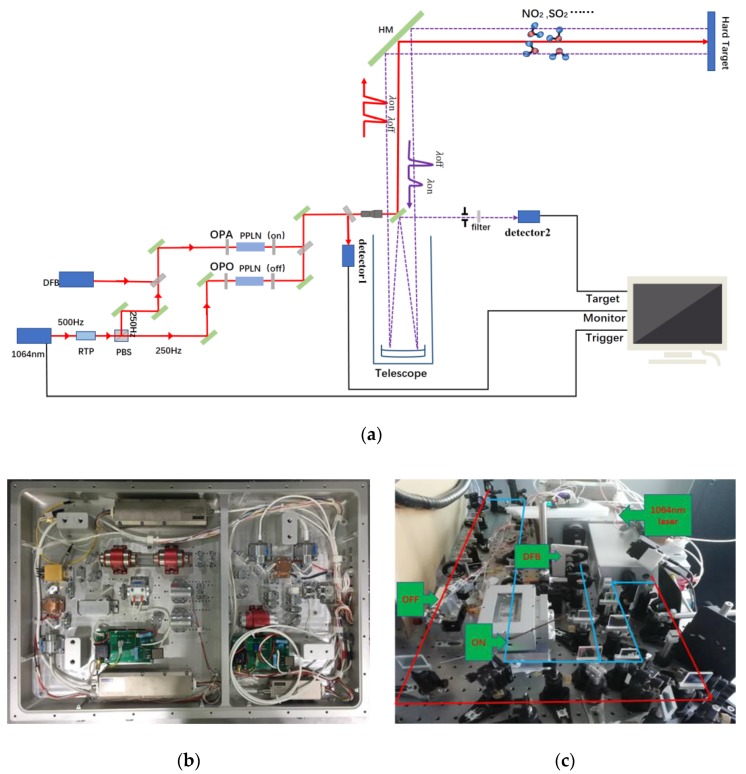
(**a)** Schematic diagram of differential absorption lidar system; (**b**) Photograph of the 1064 nm Nd: YAG laser; (**c**) Photograph of DIAL.

In the transmitter of the system, we use a 1064 nm Nd: YAG laser with a repetition rate of 500 Hz as the pump light source. The 1064 nm laser is divided into two 250 Hz lasers through RTP (Rubidium Titanyl Phosphor) crystal and PBS (Polarizing Beam Splitter) mirror. Then through their respective PPLN (Periodically Poled Lithium Niobate) channels, the 1064 nm laser is converted into mid-infrared λ_on_ laser with a DFB (Distributed Feedback Laser) injected and λ_off_ laser. By changing the PPLN crystals and adjusting temperature, the wavelength can be tuned to measure different gases. The time interval of λ_on_ and λ_off_ is 0.002 s, which can effectively reduce the error caused by environmental change, during laser alternating, and improve the anti-interference ability. The two lasers pass through the beam combining mirror, 45° reflector, beam expander, scanning mirror, then enter into the atmosphere. The scanning head can horizontally rotate from 0 to 345°, vertically rotate from 0 to 90°, and can scan space at a maximum speed of 6°/s. In the receiving part, the system uses a 300 mm Newton reflecting telescope to acquire the echo signal, then the signal is detected by a VIGO mid-infrared detector. The detector is very compact and reliable, which is easy to be integrated into the mounting assembly. An optical mounting assembly with XYZ translation movement has been designed to hold the detector and front-end focusing optics. The acquisition card (spectrum m2i-4960) has 16bit resolution and 60 MHz sampling speed. The sampling channel, trigger mode, frequency and storage mode can be set to realize the control of signal acquisition. Finally, the returned signal is stored in the hard disk of industrial computer. The whole system is installed in an experimental cabin. The laser is divided into two beams by a beam splitter with a splitting ratio of nine to one. The high-energy beam is used for normal detection. The low-energy beam weakens by the filter with transmittance of 0.01 then received by the detector. We estimate the initial laser energy based on the detector signal, beam splitting ratio and transmittance. As shown in Figure 1, a complete set of mid-infrared DIAL is provided and Table 1 shows various parameters of lidar.

**Figure 2:**
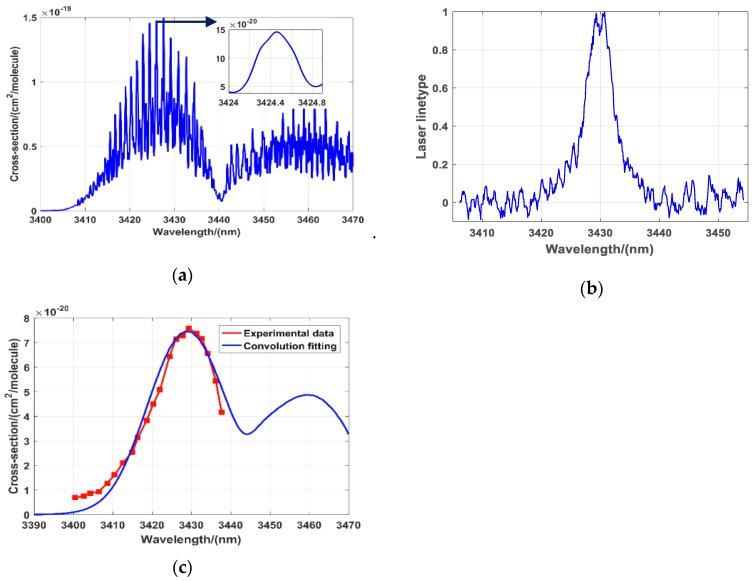
(**a**) Absorption line of NO_2_ near 3.4 μm; (**b**) Normalized laser spectrum of λ_off_; (**c**) Absorption spectral lines after convolution correction.

The wavelength of the laser should be determined prior to gas detection. Due to the injection of a DFB laser into optical parametric amplification (OPA) to obtain mid-infrared laser, and using NO_2_ as an example, the outputting λ_on_ laser spectral width is less than 0.02 nm. Comparing DFB is used as a seeder, λ_off_ uses the more economical OPO mode to output wide spectrum laser with the laser spectral width is about 10 nm. In order to evaluate the absorption of λ_off_, we use a spectrometer (YOKOGAWA, AQ6370C) to measure the laser spectral width, and then convolute the laser spectrum with the ideal gas absorption spectrum to obtain the corresponding absorption cross-section under the laser spectral width. In the process of correction, the spectral lines of laser are normalized and fitted. After convolution correction, the absorption cross-section changes obviously, but it doesn’t affect the usage. Figure 2a is the absorption spectral lines of NO_2_ near 3.4 μm from HITRAN database; (b) is the normalization laser spectrum of λ_off_; (c) is the absorption spectral lines after convolution correction. In the Figure 2c, the blue solid line is the convoluted correcting data, the red dotted solid line is the measured data of wide spectrum. The convoluted absorption cross-section no longer has a fine peak and valley structure, it presents a large absorption peak as a whole. The position of NO_2_ without absorption also changes. In order to verify the effect of the convolution correction more intuitively, we used laser spectrum at the absorption peak (3430 nm) for convolution correction, so the correction effect around 3430 nm was the best. When the wavelength was far from 3430 nm, due to slight differences in laser linewidth, there were some differences between the measured value and the calculated value. Therefore, when we selected the λ_off_ wavelength, the laser spectrum at the valley of the gas absorption spectrum was used to make precise correction. According to the result, the absorption cross-section at 3405 nm is 8.9 × 10^−21^ cm^2^/molecule, which is much less than the absorption cross-section at λ_on_ (3424.5 nm). According to the principle of wavelength optimization, the wavelength of λ_off_ is chosen at 3405 nm. The wavelength of λ_on_ laser is chosen at 3424.5 nm as shown in the Figure 2a, which is the absorption peak of NO_2_ according to the data in the HITRAN [22]. The selection of laser wavelengths λ_on_ and λ_off_ for other gases measurement is similar.

## 4. DIAL Detection Experiments and Results

The lidar system calibration experiment and open field observation experiment have been carried out in order to characterize the performance of lidar system. It is necessary to discuss the influence of gases in the environment before the experiment. The ambient concentration of SO_2_ and NO_2_ is about tens of micrograms per cubic meter, which is much smaller than emissions of pollutants from various factories. According to Lambert-Beer law:

(5) $$I_{\left(v\right)}=I_{0}exp\left[-\sigma \left(v\right)CL\right].\;$$

where $I_{0}$ is the initial laser energy, $I_{\left(v\right)}$ is the laser energy after absorption, *L* is the length of transmission path, *C* is the concentration of gas. We calculate that the attenuation due to the absorption of background gas is approximately 0.008. The ambient concentration of SO_2_ and NO_2_ has little effect on the experimental results. Before each experiment, we will also receive a set of background data to normalized correct the laser signal to counteract the effects of various gases in the atmosphere.

### 4.1. Calibration Experiment of DIAL

After constructing the DIAL system, the calibration experiment should be carried out to verify the ability of the system measuring the gas. Limited by the weather and the surrounding environment, there are few chances for lidar to observe the gas emission. At the same time, in the field experiment, the concentration of gas is easily interfered by human activities and air flow, resulting in uneven concentration distribution, and it is very difficult to verify the accuracy of lidar in an open field. In this paper, we used a set of precision calibration device to verify the accuracy of lidar detection. We simulated the equivalent gas integration path absorption in the open field by controlling the temperature and air pressure of the gas cell. In this experiment, there are no influences from field test and environmental factors that need to be considered.

**Figure 3:**
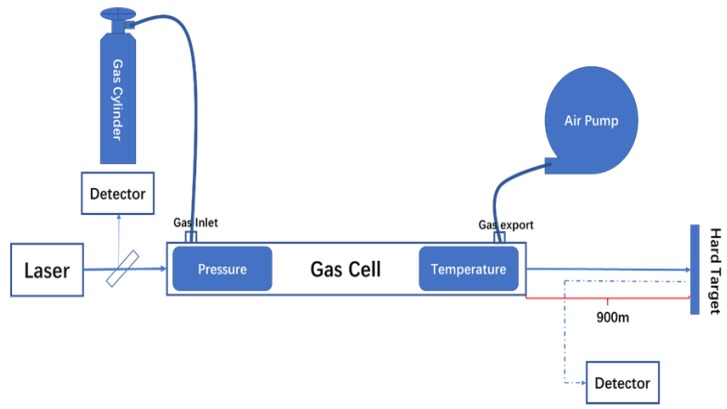
The schematic diagram of the calibration experiment.

As shown in Figure 3, the experiment used gw-1000c short optical path gas cell, with a total length of 20 cm and a diameter of 2 cm. The laser was directed to go straight through the cell, which was suitable for the spectral band of 700–12,000 nm. The gas cell was equipped with a temperature controller and a dp-100 Panasonic pressure sensor to display the real-time temperature and pressure. The experiments used 12,000 ppm NO_2_ standard gas (98.8% N_2_, 1.2% NO_2_) and 12,000 ppm SO_2_ standard gas (98.8% N_2_, 1.2% SO_2_) as the experimental gases. At the same time, in order to improve the signal-to-noise ratio, all of the λ_on_ and λ_off_ signals acquired were made up of 250 pulses on average (the corresponding acquisition time was 1 s). We took a wall with a distance of 900 m as the hard target. Prior to the experiment, we adjusted the direction of the scanner to make the laser illuminate the hard target through the atmosphere. During the experiment, we used the vacuum pump to draw out the gas in the cell, put the cell into the optical path and recorded the two detector signals as the background data. Then, we opened the standard gas cylinder and injected a proper amount of standard gas into the cell. The concentration of the gas can be calculated from the pressure and temperature of the cell. We acquired the data of the detectors under different pressures with same temperature, inverted the concentration in the gas cell and compared it with the theoretical concentration to verify the detection ability of the system.

**Figure 4:**
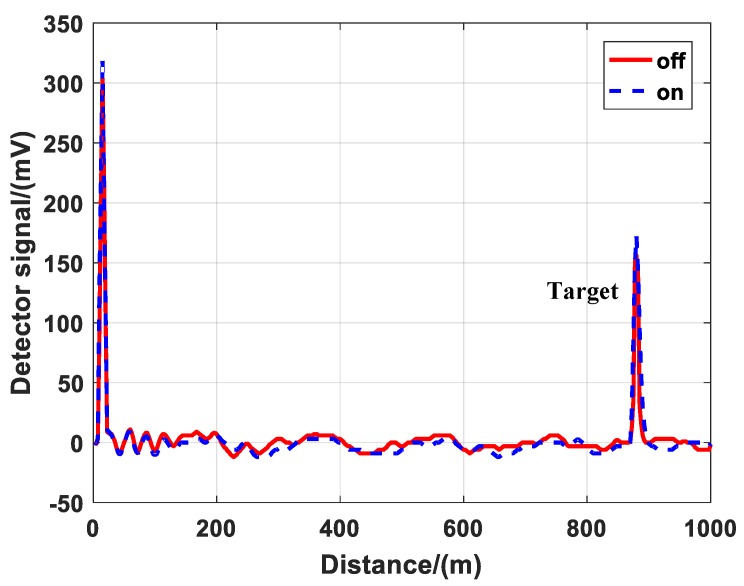
Corrected detector signals from the atmosphere and hard target background condition.

The echo signals of λ_on_ and λ_off_ were different when the cell was under the vacuum condition. Although both λ_on_ and λ_off_ lasers were powered by the same pump source, but they passed through different PPLN crystals and different optical paths, resulting in a little difference in divergence angle, energy and shape. It was difficult to ensure that the parameters of the two lasers were the same except for the wavelength. Thus, the monitoring channel was introduced. It could acquire the energy of the lidar and correct the echo signals. Figure 4 is the corrected detector signals. After the correction, we could inverse gas concentration in the next step.

We then carried out the calibration experiment. First, we opened the gas cylinder valve and emitted the standard gas into the cell. Second, the pressure in the gas cell was changed to control the absolute content of gas and the data were acquired under the different contents. Third, we calculated the concentration of gas and compared with the theoretical concentration. The experiment was carried out in the normal atmospheric environment. The temperature was maintained at 298 K and was not changing significantly, but the pressure changed by a large amount. The absorption spectrum would be greatly different under different pressures, which might increase the inversion error. According to the data of HITRAN, we corrected the ∆σ to increase the accuracy. The corrected ∆σ was substituted into Formula (3) to calculate the gas concentration as the measured result, then we used the ideal gas state equation to obtain the theoretical concentration:

(6) $$N_{all}=\frac{PV}{RT}.$$

where *P* is the pressure of the gas cell, *V* is the volume of the gas cell, *R* is the ideal gas constant, *N_all_* is the total gas molecular in the absorption cell. According to the percentage of gas in the standard gas cylinder, we got accurate concentration. We verified the detection performance of the DIAL system by comparing it with the measured results.

**Figure 5:**
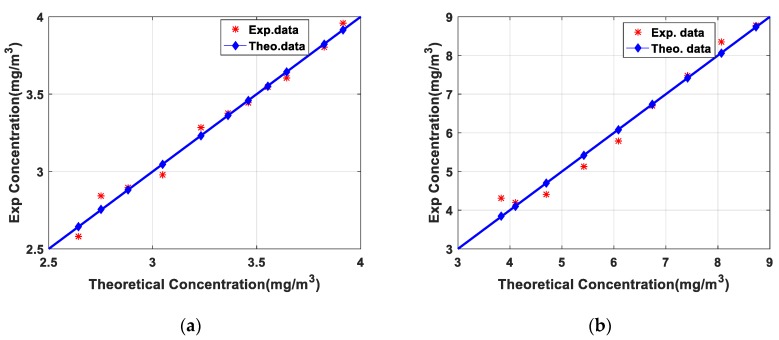
(**a**) Result of NO_2_ calibration experiment; (**b**) Result of SO_2_ calibration experiment.

Figure 5 shows the comparison results between the measured concentration from Formula (3) and the theoretical concentration in the gas cell from ideal gas state equation. The measured NO_2_ and SO_2_ gas concentrations coincided well with the theoretical concentration curve, and the inversion results were in good agreement with the theoretical results. However, there were also some differences between the measured concentration and the theoretical concentration especially at low concentrations. The gas cell cannot be completely sealed. When the gas concentration in the cell is lower, the external gas is easier to enter the cell and the error of gas inversion results is larger. In order to evaluate the matching degree between the inversion result and the theoretical value, Pearson correlation coefficient (CC) was used to evaluate the consistency and the root mean square error (RMSE) was used to evaluate the deviation. The CC and RMSE of NO_2_ were 0.993 and 0.047 respectively while the CC and RMSE of SO_2_ were 0.988 and 0.2582. The calibration experiments of NO_2_ and SO_2_ ensured the reliability of the lidar system and the inversion algorithm.

### 4.2. Open Field Observation of DIAL

**Figure 6:**
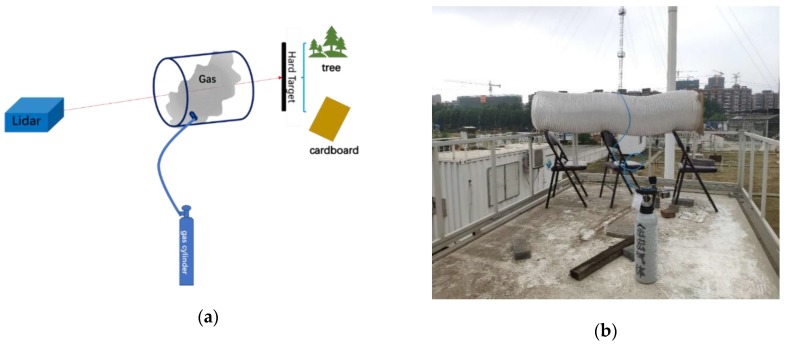
(**a**) Schematic diagram of open field gas measuring experiment; (**b**) Photograph of open field gas measuring experiment.

In order to explore the detection capability of gas emission in the open field, we carried out the detection of polluted gas emissions in open field during the afternoon of 21 May 2019. The meteorological condition is relative stable and the atmospheric temperature was 24 °C, the relative humidity was 42%, visibility was 26 km and windspeed was 3 m/s. Figure 6a shows the schematic diagram of open field gas measuring experiment and Figure 6b shows the photograph of open field gas measuring experiment. We placed an exhaust pipe with length of 2 m and diameter of 400 mm at a distance of 200 m from the lidar system. The two ends of the pipe were open while the sidewall was sealed. A circular vent was left on the sidewall to connect with standard gas cylinder to simulate the discharge of pollution gases. Prior to the experiment, the scanning head of lidar was pointed to the pipe to ensure that the laser passed through the pipe completely and reached the hard target. In the infrared spectrum, the contribution of molecular scattering to the total signal was negligible. The returned signal depended on the reflected signal of the hard target and the pollution gas column content. The differential absorption signal ratio was calculated according to the returned signal from the hard target and then the gas concentration was inverted. In order to make the results repeatable and nonspecific, the experiments were carried out with trees, and board, as hard targets respectively. The board was directly placed on the rear end of the pipe. The trees were the background targets located at the rear end of the pipe. In the experiment, we firstly operated the lidar to collect the background signal of the system, then opened the cylinder valve releasing the gas to the pipe. When the concentration reached the maximum, we closed the cylinder valve and waited for the gas to dissipate. The system acquired the signal intensity of the whole release process and inverted the concentration of gases. 

**Figure 7:**
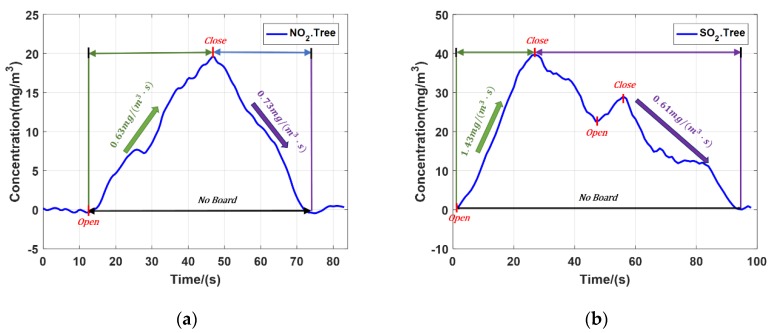
(**a**) Average concentration of NO_2_; (**b**) Average concentration of SO_2_.

The experiments of NO_2_ and SO_2_ emission were carried out with the trees as the hard target. Based on the characteristics of high repetition rate and signal-to-noise ratio, the temporal resolution can reach second level. Figure 7a is average concentration of NO_2_ and Figure 7b is average concentration of SO_2_. The two experiments completely recorded the changes of NO_2_ and SO_2_ concentration. During the NO_2_ experiment, the gas cylinder was opened once. When the cylinder opened, the NO_2_ concentration rose at the speed 0.63 mg/(m^3^ s). Then we closed the cylinder, the gas concentration dropped at the speed of 0.73 mg/(m^3^·s). Finally, all NO_2_ dissipated and the concentration returned to the initial concentration value. During the SO_2_ experiment, the gas cylinder was opened twice. The valve was fully opened in the first time, and then the valve was half opened, the second time. During the first opening of the valve, the average SO_2_ concentration rose sharply at the speed of 1.43 mg/(m^3^·s). Then the valve was closed, the gas concentration decreased at the speed of 0.61 mg/(m^3^·s). Before the dissipation of the SO_2_, the valve was opened again and the SO_2_ concentration increased. Finally, all SO_2_ dissipated and the concentration returned to the initial value.

**Figure 8:**
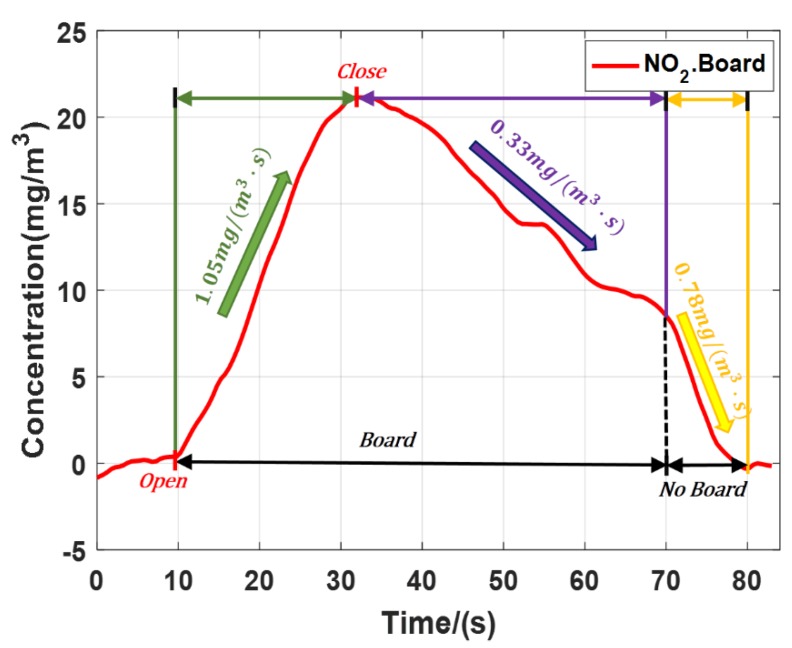
Average concentration of NO_2_ with board as the hard target.

The NO_2_ emission experiment was carried out with a board as the hard target. Figure 8 shows the result. When the gas cylinder valve was half opened, the NO_2_ concentration rose at the speed of 1.05 mg/(m^3^·s), Then we closed the gas cylinder valve, the NO_2_ concentration slowly dropped at the speed of 0.33 mg/(m^3^·s). After a period of time, we removed the board and the gas dissipation speed increased to 0.78 mg/(m^3^·s), basically consistent with the gas dissipation speed in Figure 7a. Because the board was directly placed behind the pipe, it was not easy for the gas to flow out from the pipe. Compared with the results of experiment which taking the trees as the hard target, the board just behind the pipe kept the high concentration of NO_2_ in the pipe. The experimental results were in line with the scientific reality. According to *SNR* of lidar and ∆σ of polluted gases, substituting Formula (4) to calculate, the minimum detection sensitivity was 0.07 mg/m^3^ (NO_2_) and 0.31 mg/m^3^ (SO_2_).

## 5. Conclusions

In this work, a set of 2.5–4 μm solid-state high repetition rate mid-infrared DIAL has been developed. By changing the PPLN crystals and adjusting the temperature, the wavelength can be tuned to measure a variety of gases. In this study, taking NO_2_ and SO_2_ as examples, the original inversion equation is modified to correct the system error caused by the lidar system. We carried out the lidar system calibration experiment and open field observation experiment. The calibration experiment showed CC was higher than 98%, which ensured the lidar’s detection ability for measurement of the pollution gases. The open field observation experiment showed the lidar system can record the real-time concentration changes of gas with high temporal resolution up to second level. The minimum detection sensitivity for NO_2_ and SO_2_ was 0.07 mg/m^3^, and 0.31 mg/m^3^, respectively. The system has advantages in temporal resolution, which can be used to monitor the concentration of pollution gases around plants and help improve the early warning tasks. Using a pipe to simulate gas emission in the open field is not an ideal approach. In the next stage of research, we will use the system to detect the gas emission of chemical plants to verify the detection performance in a real open field. We will continue to make full use of the characteristics of wavelength tunable and high temporal resolution to realize the effective detection of some other kinds of gases.

## Figures and Tables

**Table 1:** Main Performance Parameter of the lidar.

Typical Parameter	Value	
Wavelength	λ_on_/ λ_off_(NO_2_)	3424.5/3405 nm
	λ_on_/ λ_off_(SO_2_)	3988.9/3940.0 nm
	λ_on_/ λ_off_(NO)	2657.4/2630.4 nm
Pulse energy	0.14 mJ/0.12 mJ	
Pulse duration	20 ns	
Pulse Linewidth	0.02 nm(on)/10 nm(off)	
Pulse rate (on/off)	250 Hz/250 Hz	
Sampling rate	60 MHz	
Sampling digit	16 bit	
Detector bandwidth	5 MHz	
Detector sensitivity	6.5 × 10^4^ V/W	
Detector spectral response range	2.5–4.5 μm	
